# The endothelial glycocalyx and its disruption, protection and regeneration: a narrative review

**DOI:** 10.1186/s13049-016-0239-y

**Published:** 2016-04-12

**Authors:** Ulf Schött, Cristina Solomon, Dietmar Fries, Peter Bentzer

**Affiliations:** Department of Clinical Sciences Lund, Medical Faculty, University of Lund, Lund, Sweden; Department of Intensive and Perioperative Care, Skane University Hospital, Lund, Sweden; Department of Anesthesiology, Perioperative Medicine and General Intensive Care, Paracelsus Medical University, Salzburg, Austria; CSL Behring, Marburg, Germany; Department of Surgical and General Critical Care Medicine, Medical University Innsbruck, Innsbruck, Austria

**Keywords:** Bleeding management, Fresh frozen plasma, Glycocalyx, Protection, Regeneration

## Abstract

The glycocalyx is a carbohydrate-rich layer that lines the luminal side of the vascular endothelium. Its soluble components exist in a dynamic equilibrium with the bloodstream and play an important role in maintaining endothelial layer integrity. However, the glycocalyx can be easily damaged and is extremely vulnerable to insults from a variety of sources, including inflammation, trauma, haemorrhagic shock, hypovolemia and ischaemia-reperfusion. Damage to the glycocalyx commonly precedes further damage to the vascular endothelium. Preclinical research has identified a number of different factors capable of protecting or regenerating the glycocalyx. Initial investigations suggest that plasma may convey protective and regenerative effects. However, it remains unclear which exact components or properties of plasma are responsible for this protective effect. Studies have reported protective effects for several plasma proteins individually, including antithrombin, orosomucoid and albumin; the latter of which may be of particular interest, due to the high levels of albumin present in plasma. A further possibility is that plasma is simply a better intravascular volume expander than other resuscitation fluids. It has also been proposed that the protective effects are mediated indirectly via plasma resuscitation-induced changes in gene expression. Further work is needed to determine the importance of specific plasma proteins or other factors for glycocalyx protection, particularly in a clinical setting.

## Background

The endothelial glycocalyx is a carbohydrate-rich layer that lines the vascular endothelium. The presence of a protein layer on the endothelium was first proposed by Danielli in 1940 [1] and was visualised using electron microscopy in 1966 [2]. Initial investigations of the glycocalyx were hampered as previous staining and fixing techniques destroyed this fragile structure [3]. However, contemporary methods preserve the glycocalyx and have enabled more detailed examination of the structure and physiology of this layer [4].

**Fig. 1:**
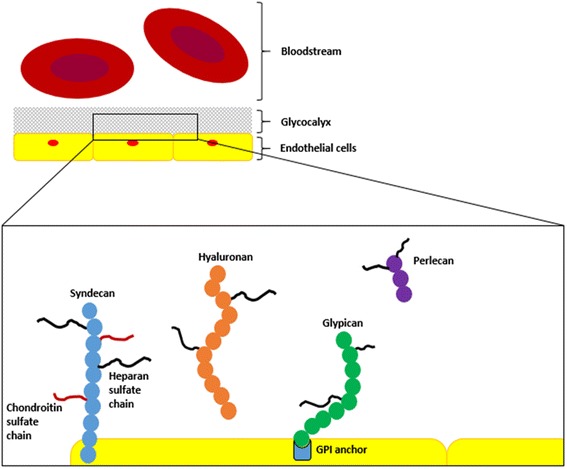
Schematic representation of the glycocalyx and its constituents. Syndecan and glypican are membrane-bound proteoglycans which carry chondroitin sulfate and heparan sulfate side chains. Syndecans are anchored to the plasma membrane via a single-span transmembrane protein and may therefore act as receptors. Glypicans are attached to the cell surface via a glycosylphosphatidylinositol (GPI) anchor. The glycocalyx is also comprised of secreted proteoglycans, such as perlecan, together with secreted glycosaminoglycans, such as hyaluronan. Note that this figure is not drawn to scale

The glycocalyx is connected to the endothelium via several cell-bound core molecules, mainly proteoglycans and glycoproteins (Fig. 1). On the luminal surface, the glycocalyx is formed by soluble plasma components, either linked to each other directly or via soluble proteoglycans, glycosaminoglycans and sialoproteins [5, 6]. A dynamic equilibrium exists between the layer of soluble components and the bloodstream, where the blood flow constantly affects both the composition and the thickness of the glycocalyx [5]. For example, Ueda and colleagues reported that the glycocalyx in bovine aortic endothelial cells increase in thickness as sheer stress increases up to 3.0 Pa [7]. Additionally, the glycocalyx contains a large volume of non-circulating plasma; estimated at 1–1.7 L [8, 9], and has a net negative charge that affects its interaction with plasma constituents, platelets and red blood cells, thereby counteracting microvascular thrombosis and maintaining rheology [4, 10, 11].

Situated between the endothelium and the bloodstream, the glycocalyx serves several functions including limiting access of particular molecules to the endothelium, influencing blood cell–vessel wall interactions and acting as a mechanotransducer (Table 1) [5, 10, 12–20]. For example, the glycocalyx is semipermeable to certain macromolecules such as plasma proteins (e.g. albumin) but cannot be penetrated by red blood cells or large molecules such as dextran (>70 kD) [16]. Conformational changes in glycocalyx structure also lead to release of nitric oxide, affecting vasomotor tone and tissue perfusion [18]. Furthermore, as the glycocalyx binds plasma proteins it creates a high intravascular oncotic pressure at the endothelial surface; The net outward movement of fluid is therefore opposed by this inward-directed oncotic pressure, according to the Starling equation and the paracellular permeability model [21].

**Table 1:** Functions of the glycocalyx

Function	Reference
Forms the interface between the vessel wall and the bloodstream	Reitsma et al. 2007 [5]
	Alphonsus and Rodseth 2014 [10]
Maintains the colloid osmotic gradient of the vascular barrier	Rehm et al. 2007 [12]
Acts as a barrier to:	
vascular exchange of water and solutes	Reitsma et al. 2007 [5]
leukocyte–endothelium adhesion	Henry and Duling 1999 [13]
	Lipowsky et al. 2011 [14]
	Constantinescu et al. 2003 [15]
	Becker et al. 2015 [19]
Acts as a sieve for plasma proteins	Vink and Duling 2000 [16]
	Lipowsky et al. 2011 [14]
Provides binding sites for everal molecules, including:	
antithrombin III	Alphonsus and Rodseth 2014 [10]
tissue factor pathway inhibitors	Reitsma et al. 2007 [5]
lipoprotein lipase	Kolářová et al. 2014 [18]
vascular endothelial growth factor	
fibroblast growth factor	
extracellular superoxide dismutase	
hyaluronic acid molecules	
Acts as a shear stress sensor and regulator of mechanotransduction	Reitsma et al. 2007 [5]
	Florian et al. 2003 [17]
	Yen et al. 2014 [20]
	Becker et al. 2015 [19]

Other soluble components that bind to the luminal portions of the glycocalyx are unbound hyaluronic acid molecules, superoxide dismutase, antithrombin III, protein C and cell adhesion molecules [18]. Due to the many functions of the glycocalyx, it is perhaps unsurprising that any disruption or damage to this layer contributes towards numerous vascular pathologies [5].

### Literature review

The aim of this paper was to review and summarise the clinical findings relating to the biological functions of the glycocalyx layer and its damage and regeneration or protection. As a number of publications were found to evaluate the restorative effects of fresh frozen plasma (FFP), this review goes on to explore if a specific component of FFP is considered to be responsible for these effects. A non-systematic literature search was conducted using MEDLINE (PubMed) with the objective of identifying English language articles published up to 14^th^ January 2016, which provided information regarding the endothelial glycocalyx and its functions. A broad search was performed using the term “glycocalyx” and a total of 2458 articles were retrieved. Following review of the title and abstract, 2318 articles were discarded as they were not relevant e.g. non-English language, not related to damage or protection/regeneration. Reviews and articles that described general roles and structure of the glycocalyx were retained for background information. Finally, additional articles of interest that were identified during development of this review were also included.

### Damage to the glycocalyx

The glycocalyx is surprisingly delicate and sustains injury quite easily. Damage can range from deterioration through to fundamental destruction of the glycocalyx. Studies in isolated organs, animal models and clinical studies have investigated how the glycocalyx is damaged and this research is described here.

#### In vitro and isolated organ models

Studies in isolated organs and cells demonstrate that disruption and shedding of the glycocalyx is associated with a number of different molecules and stress factors. Topical and intrascrotal application of TNF-α to the hamster cremaster microcirculation resulted in damage to the glycocalyx, as observed by increased access of fluorescein isothiocyanate (FITC)-labeled dextrans 70 and 580 to the space bounded by the apical glycocalyx in arterioles, capillaries and venules [22]. Atrial natriuretic peptide was also shown to be associated with shedding of the glycocalyx, assessed by increased release of syndecan-1 in the coronary vascular bed in a guinea pig heart model [23]. Furthermore, abnormal blood shear stress was also shown to increase glycocalyx shedding in cultured human vascular umbilical endothelial cells [24].

Shedding of the glycocalyx components appears to be a regulated process influenced by several factors. A study in SVEC4-10 endothelial cells demonstrated increased glycocalyx shedding in response to a range of stimuli including the proteases thrombin and plasmin, as well as the protein kinase C modulator phorbol 12-myristate 12-acetate and epidermal growth factor, suggesting shedding is also modulated by tyrosine kinases and G protein-coupled receptors (GPCRs), in addition to proteases [25].

#### Animal models

In rodent models, disruption and shedding of the glycocalyx components, like syndecan-1 and heparan sulfate, has been demonstrated in response to hyperglycaemia [26], haemorrhagic shock [27, 28], inflammation and ischaemia-reperfusion injury [29]. Increased shedding and decreased colloid osmotic pressure (COP) could be markers of inflammation [28], but other factors may be equally responsible for the observed effects on both glycocalyx shedding and endothelial permeability.

In a porcine model, the combination of traumatic brain injury and haemorrhagic shock resulted in glycocalyx shedding within 15 min after injury [30].

#### Clinical studies

Shedding of the glycocalyx in humans has been demonstrated in response to hypovolaemia [31] and has also been observed in patients undergoing aortic or cardiac bypass surgery [12, 32]. In patients undergoing aortic bypass surgery, it was demonstrated that heparan sulfates shed from the glycocalyx can directly activate leukocytes and platelets which, in turn, release heparanases that can also degrade heparan sulfate chains, leading to a vicious cycle that can make it challenging to confirm the initial cause of the glycocalyx damage [12]. Thus, in line with data obtained from experiments in isolated organs and animal models, disruption of the glycocalyx in humans also leads to the release of specific components into the circulation. In a clinical setting, these components may be valuable in assessing the severity of the damage and clinical outcomes. Circulating levels of syndecan-1 and heparan sulfate are proportionally related to the degree of glycocalyx damage. For example, in trauma patients, a high level of syndecan-1 is associated with coagulopathy and increased mortality [33] and, in patients with septic shock, increased levels of glycosaminoglycans have been shown to correlate with mortality [34]. Rahbar and colleagues [35] observed shedding of four primary components of the glycocalyx (syndecan-1, heparan sulfate, hyaluronic acid and chondroitin sulfate) in severely injured trauma patients and noted that syndecan-1, heparan sulfate and endothelial cell permeability were significantly higher in patients with reduced plasma COP; thrombin generation was also impaired in these patients. The loss of thrombin generation may be explained by endogenous heparinisation as suggested by Ostrowski and Johansson [36]. However, further studies are warranted before drawing definitive conclusions regarding any implied causality between glycocalyx degradation and decreased COP. In a study by Koning and colleagues, patients undergoing cardiac surgery with cardiopulmonary bypass (CPB) experienced acute glycocalyx injury in contrast to off-pump surgery [32]. Glycocalyx shedding is also linked to endothelial cell damage, often measured as high levels of soluble thrombomodulin (sTM) [36]. sTM is an endothelial cell transmembrane glycoprotein that is only released upon direct endothelial disruption. In sepsis, sTM better predicted risk of multiple organ failure compared with syndecan-1, and only sTM predicted death [37]. Additionally, syndecan-1 measured during emergency department admission has been found to be useful for assessing the risk of developing acute kidney injury and in-hospital mortality in patients with acute decompensated heart failure [38]. Based on these findings, further investigations of whether specific markers of glycocalyx integrity may be of value in guiding treatment and clinical decision-making may be warranted.

### Protection and regeneration of the glycocalyx

Given its importance in various physiological functions and the delicate nature of this layer, protection of the glycocalyx may prove a promising target in critical care settings as well as in the treatment of chronic vascular disease. In fact, several studies have investigated whether the glycocalyx can be protected or if damage can be repaired. Some of the agents that have been studied are detailed below (Table 2).

**Table 2:** Treatments linked with protection of the glycocalyx

Treatment	Reference
Hydrocortisone	Chappell et al. 2007, 2009b, 2010 [41, 43, 44]
Antithrombin	Chappell et al. 2009a, 2009b, 2010 [42–44]
Protein C	Marechal et al. 2008 [50]
Nitric oxide	Bruegger et al. 2008 [45]
Hyaluronic acid and chondroitin sulphate	Henry and Duling 1999 [13]
Sulodexide	Broekhuizen et al. 2010 [54]
Lidoflazine	Flameng et al. 1983 [55]
Albumin	Jacob et al. 2006, 2009 [46, 47]
Hydroxethyl starch	Rehm et al. 2004; Jacob et al. 2006 [8, 46]
N-acetylcysteine	Nieuwdorp et al. 2006 [9]
Metformin	Eskens et al. 2013 [51]

#### In vitro and isolated organ models

In a study of rat fat-pad endothelial cells, glycocalyx shedding induced by removal of albumin from the culture medium, was rescued by application of sphingosine-1-phosphate, demonstrating that sphingosine-1-phosphate GPCRs are also involved in preserving glycocalyx integrity [39]. Additionally, the same group found that the role of sphingosine-1-phosphate on glycocalyx recovery is mediated by the phosphoinositide 3-kinase pathway [40].

Using isolated perfused guinea pig hearts, Chappell and colleagues demonstrated that the glycocalyx could be protected against ischaemia-reperfusion injury or inflammatory degradation initiated by TNF-α by pre-treatment with hydrocortisone or antithrombin [41–44]. Exogenous administration of nitric oxide during reperfusion was also shown to protect the glycocalyx in isolated guinea pig hearts [45]. However, nitric oxide had no preventive effect on coronary leak or oedema if the glycocalyx was destroyed enzymatically beforehand. A combination of hyaluronic acid and chondroitin sulphate, two glycocalyx abundant glycosaminoglycans, partially regenerated the capillary glycocalyx damaged by hyaluronidase in hamsters; treatment with either molecule separately had no effect [13].

In a guinea pig heart model of transplantation-induced ischaemia/reperfusion glycocalyx damage, infusion of 5 % albumin or 6 % hydroxethyl starch, a natural and an artificial colloid, led to decreased fluid extravasation [8, 46]. Augmenting the traditional preservation solution with albumin was also reported to protect the glycocalyx in this model [47]. Based on these findings, the researchers suggested that maintaining a physiological albumin blood concentration before the onset of a surgical procedure may be adequate to protect vascular integrity [46]. The effect of FFP was investigated in an in vitro model of endothelial injury and found that FFP, but not lactated Ringer’s, preserved syndecan-1 and maintained endothelial junction integrity [48]. The mechanism by which FFP mitigates syndecan-1 shedding is unknown. The authors hypothesise that FFP may inhibit or neutralise sheddases (a diverse group of proteases) and/or that FFP mobilises intracellular stores of preformed syndecans.

#### Animal models

In the postcapillary venules of the rat mesentery, glycocalyx shedding, induced by the chemoattractant f-Met-Leu-Phe, was significantly reduced by inhibition of matrix metalloproteinases using the zinc chelator ilomastat, as well as subantimicrobial concentrations of doxycycline [49]. During endotoxaemia, activated protein C prevented capillary perfusion deficit in lipopolysaccharide-treated rats leading to preservation of the glycocalyx [50]. In a mouse model of non-insulin dependent diabetes mellitus, treatment with metformin was associated with an improvement in the barrier properties of the glycocalyx [51].

Using a haemorrhagic shock model in rats, Kozar and colleagues investigated the effect of plasma resuscitation on the glycocalyx [27]. Early signs of glycocalyx restoration were demonstrated within 2 h of plasma resuscitation, compared with no repair with lactated Ringer’s solution. Furthermore, expression of lung syndecan-1 mRNA was enhanced by plasma resuscitation, suggesting that plasma restores the backbone of the glycocalyx [27]. The authors concluded that the protective effects of plasma may be due, in part, to its ability to restore the glycocalyx and preserve syndecan-1 after haemorrhagic shock.

In a haemorrhagic shock model in microvessels of the cremaster muscle, the effects of FFP, a 6 % hydroxyethyl starch solution and lactated Ringer’s solution were compared for their ability to repair the endothelial glycocalyx structure, promote volume expansion, increase blood flow and prevent coagulopathy. After resuscitation, glycocalyx thickness was reduced by approximately 70 % in rats in the haemorrhage only (no resuscitation), 6 % hydroxyethyl starch or lactated Ringer’s groups whereas the FFP-treated rats recovered glycocalyx thickness 1 h post-resuscitation [28]. Plasma syndecan-1 levels were also restored to baseline levels following resuscitation with FFP compared with the 6 % hydroxyethyl starch and lactated Ringer’s groups which were 50 % higher than sham and FFP. Furthermore, resuscitation with FFP improved coagulation and blood flow and restored pH and base excess to baseline levels. The authors state that the efficacy of FFP may be related to reconstitution of the glycocalyx structure, volume expansion and renewal of overall protein levels.

In a mouse model of haemorrhagic shock and trauma, treatment with FFP and spray-dried plasma (SDP) were compared with lactated Ringer’s solution [52]. Both FFP and SDP reduced permeability and inflammation and modulated vascular integrity. Mean arterial pressures and base excess were also corrected to levels observed in sham-treated mice by both FFP and SDP.

In a haemorrhagic shock model, rats were resuscitated with either fresh whole blood (FWB), 1:1 packed RBC (pRBC)/lactated Ringer’s, 1:1 washed pRBC/lactated Ringer’s or lactated Ringer’s alone [53]. Resuscitation with FWB and pRBCs improved glycocalyx thickness compared with washed pRBCs, suggesting that the protective effects on the endothelium may be associated with the presence of residual plasma proteins.

#### Clinical studies

In clinical trials of patients with type 2 diabetes, sulodexide administration for 2 months increased glycocalyx thickness; likely as a result of enhanced precursor abundance for glycosaminoglycan synthesis [54]. In an older study, pre-treatment with lidoflazine (a calcium channel blocker) preserved the glycocalyx in a dose-dependent manner in patients undergoing multiple aorta-coronary bypass grafting [55]. Furthermore, controlling diet-induced hyperlipidaemia and hypercholesterolaemia could also protect the glycocalyx [4].

As mentioned earlier, the glycocalyx can be damaged in a variety of clinical situations, including cardiac surgery and haemorrhagic shock. However, in patients undergoing cardiac surgery with CPB, the use of pulsatile flow was associated with recovery of glycocalyx dimensions within four hours of the initial injury caused by the onset of extracorporeal circulation; this recovery was absent after nonpulsatile CPB [32].

Haywood-Watson and colleagues investigated whether FFP would restore the glycocalyx and reduce syndecan-1 shedding in severely injured patients in haemorrhagic shock [48]. In line with data from pre-clinical experiments, plasma syndecan-1 levels were markedly elevated in these patients and correlated with specific inflammatory cytokines (IFN-ɣ, fractalkine and IL-1β were negatively correlated while IL-10 was positively correlated), all of which play an important role in maintaining the endothelial integrity [48]. Plasma syndecan-1 levels were found to decrease following resuscitation but were still elevated compared to normal patients, suggesting partial restoration of the glycocalyx took place following administration of FFP. Furthermore, it has been shown that plasma-based resuscitation of trauma patients in haemorrhagic shock leads to a decrease in mortality [56, 57].

### Which factors mediate the restorative effects of plasma?

The above studies indicate that FFP has beneficial effects on the glycocalyx. These benefits have been shown to diminish once FFP has been thawed and then stored before use [58]. In vitro studies have shown that FFP decrease the permeability of pulmonary endothelial cells. This effect was diminished when FFP was thawed and stored for 5 days compared with freshly thawed FFP; FFP stored for 10 days had no protective effect and actually exacerbated permeability. Additionally, the thrombin generating capacity of FFP was also found to diminish between Days 0 and 5 of storage, and in vivo studies demonstrated that Day 0 FFP was able to restore and maintain blood pressure at baseline levels whereas Day 5 could not [58].

The question that remains to be answered is what specific constituents or properties of the plasma are mediating its protective effect. Several factors may be involved in mediating the restorative effects of plasma on the glycocalyx. One possibility is that plasma is simply a better volume expander compared to crystalloid resuscitation fluids and therefore more effectively counteracts ischemia-induced changes of the glycocalyx. Another possibility is that alterations in gene expression and protein synthesis in response to plasma resuscitation play a vital role. Finally, it is possible that one or more plasma proteins are directly responsible for the protective effects. For instance, plasma albumin constitutes the largest pool of thiols in the circulation and effectively reduces oxidative stress due to its radical-scavenging reduced cysteine and methionine residues [59]. Protective effects may also be mediated by the ability of albumin to inhibit platelet activation [60] or its ability to transport a variety of free fatty acids [61] and hormones [62].

#### In vitro and isolated organ models

It is likely that specific plasma proteins are involved in glycocalyx protection and regeneration, and data from isolated organ models have identified several compounds capable of either protecting or damaging the glycocalyx. Protective effects have been reported for antithrombin III in an isolated guinea pig heart model [42]. Conversely, C-reactive protein dose-dependently increased hyaluronan while decreasing heparan sulfate surface expression and glycocalyx thickness in human aortic endothelial cells [63]. A recent study by Deng and colleagues found that immunodepletion of adiponectin from FFP eliminated its ability to inhibit hypoxia-induced hyperpermeability in human endothelial cells [64].

#### Animal models

Adiponectin has been found to be an important component of FFP in a mouse model of haemorrhagic shock [64]. In this study, immunodepletion of adiponectin from FFP eliminated its effects on improving lung vascular barrier function in mice after haemorrhagic shock; however, replenishment with FFP rescued these effects.

In studies where the proposed beneficial effects of FFP have been demonstrated, plasma volume has not been measured, and it is not known whether comparable volume expansion was achieved between the treatment groups. Comparable volume expansion was the objective in an in vivo study employing a mouse trauma and haemorrhagic shock model where FFP and crystalloids were used at a ratio of 1:3 [65]. FFP was shown to restore the glycocalyx and reduce syndecan-1 shedding. However, since plasma volumes were not measured it is not known if this effect was due to improved volume expansion or other properties of plasma [65].

In order to determine whether the protective effects of plasma are secondary to better volume expansion compared to crystalloids, we recently investigated the effects of resuscitation with FFP or Ringer’s acetate on plasma volume expansion and glycocalyx shedding in a rat model of haemorrhagic shock. The ratio of FFP to Ringer’s acetate in this study was ≈ 1:4. Even so, FFP produced a greater plasma volume expansion than Ringer’s acetate, suggesting that FFP is a better plasma volume expander than previously thought [66]. Although we did not discover any evidence of FFP-mediated reduction in glycocalyx shedding in this study it should be noted that degradation markers were only assessed at one time point, exactly two hours after resuscitation. It is possible that differences between FFP and other treatments are detectable at other time points.

Another possibility is that protective effects are mediated indirectly, via plasma resuscitation-induced changes in gene expression. For instance, Potter and colleagues demonstrated that FFP and SDP, but not lactated Ringer’s, conveyed endothelial cell protection and induced a similar pattern of endothelial cell gene expression in a murine model of haemorrhagic shock [52]. These data suggest that plasma may control cell–cell communication and vasoreactivity of the endothelium, and may therefore serve as a starting point for elucidating the mechanism of action of plasma on the glycocalyx.

#### Clinical studies

Evidence of the protective effects of plasma is not limited to pre-clinical data. A prospective, observational study in severely injured patients with haemorrhagic shock demonstrated significantly elevated levels of syndecan-1 (554 ± 93 ng/ml) in injured patients [48]. Resuscitation with FFP resulted in an approximately 3-fold decrease in circulating syndecan-1 levels (187 ± 36 ng.ml), although they remained elevated compared to the levels measured in a control group of healthy plasma donors (27 ± 1 ng/ml).

In a study of non-bleeding, critically ill patients, a prophylactic dose of FFP was infused prior to an invasive procedure [67]. FFP transfusion decreased subsequent levels of syndecan-1 and was associated with an increase in the von Willebrand factor (vWF)-cleaving metalloproteinase ADAMTS13 and a corresponding decrease in vWF. Large vWF multimers damage the endothelium and the authors suggest that ADAMTS13 may have increased the ability to cleave vWF large multimers present on the endothelium, potentially stabilising the condition of the endothelium. Notably, this appears to contradict results from the animal studies [49] which suggested that inhibition of metalloproteases with hydroxamate inhibitors, known to inhibit ADAMTS13 [68], reduce glycocalyx shedding.

It is worth noting that plasma contains an abundance of proteins, and it remains unclear to what extent one specific protein showing benefits in isolation is involved in the protective effects of plasma in vivo, and by which mechanism.

## Conclusions

The glycocalyx plays a fundamental role in the microcirculation and in the initiation and regulation of coagulation and inflammation. Therapeutic strategies aimed at protecting this delicate layer are poorly understood at this time. While the various beneficial effects of FFP have been demonstrated, there is still no information as to what the mode of action is and if this is due to a particular constituent of the plasma. Further research is necessary to understand how FFP and other therapeutic strategies can benefit protect the glycocalyx and ultimately the patient.

## Abbreviations

COP: colloid osmotic pressure
FFP: fresh frozen plasma
GPCR: G protein-coupled receptor
SDP: spray-dried plasma
sTM: soluble thrombomodulin
vWF: von Willebrand factor

## References

[1] Danielli JF (1940). Capillary permeability and oedema in the perfused frog. J Physiol.

[2] Luft JH (1966). Fine structures of capillary and endocapillary layer as revealed by ruthenium red. Fed Proc.

[3] Pries AR, Secomb TW, Gaehtgens P (2000). The endothelial surface layer. Pflugers Arch.

[4] Chelazzi C, Villa G, Mancinelli P, De Gaudio AR, Adembri C (2015). Glycocalyx and sepsis-induced alterations in vascular permeability. Crit Care.

[5] Reitsma S, Slaaf DW, Vink H, van Zandvoort MA, oude Egbrink MG (2007). The endothelial glycocalyx: composition, functions, and visualization. Pflugers Arch.

[6] Li L, Ly M, Linhardt RJ (2012). Proteoglycan sequence. Mol Biosyst.

[7] Ueda A, Shimomura M, Ikeda M, Yamaguchi R, Tanishita K (2004). Effect of glycocalyx on shear-dependent albumin uptake in endothelial cells. Am J Physiol Heart Circ Physiol.

[8] Rehm M, Zahler S, Lotsch M, Welsch U, Conzen P, Jacob M (2004). Endothelial glycocalyx as an additional barrier determining extravasation of 6 % hydroxyethyl starch or 5 % albumin solutions in the coronary vascular bed. Anesthesiology.

[9] Nieuwdorp M, van Haeften TW, Gouverneur MC, Mooij HL, van Lieshout MH, Levi M (2006). Loss of endothelial glycocalyx during acute hyperglycemia coincides with endothelial dysfunction and coagulation activation in vivo. Diabetes.

[10] Alphonsus CS, Rodseth RN (2014). The endothelial glycocalyx: a review of the vascular barrier. Anaesthesia.

[11] Bansch P, Nelson A, Ohlsson T, Bentzer P (2011). Effect of charge on microvascular permeability in early experimental sepsis in the rat. Microvasc Res.

[12] Rehm M, Bruegger D, Christ F, Conzen P, Thiel M, Jacob M (2007). Shedding of the endothelial glycocalyx in patients undergoing major vascular surgery with global and regional ischemia. Circulation.

[13] Henry CB, Duling BR (1999). Permeation of the luminal capillary glycocalyx is determined by hyaluronan. Am J Physiol.

[14] Lipowsky HH, Gao L, Lescanic A (2011). Shedding of the endothelial glycocalyx in arterioles, capillaries, and venules and its effect on capillary hemodynamics during inflammation. Am J Physiol Heart Circ Physiol.

[15] Constantinescu AA, Vink H, Spaan JA (2003). Endothelial cell glycocalyx modulates immobilization of leukocytes at the endothelial surface. Arterioscler Thromb Vasc Biol.

[16] Vink H, Duling BR (2000). Capillary endothelial surface layer selectively reduces plasma solute distribution volume. Am J Physiol Heart Circ Physiol.

[17] Florian JA, Kosky JR, Ainslie K, Pang Z, Dull RO, Tarbell JM (2003). Heparan sulfate proteoglycan is a mechanosensor on endothelial cells. Circ Res.

[18] Kolarova H, Ambruzova B, Svihalkova Sindlerova L, Klinke A, Kubala L (2014). Modulation of endothelial glycocalyx structure under inflammatory conditions. Mediators Inflamm.

[19] Becker BF, Jacob M, Leipert S, Salmon AH, Chappell D (2015). Degradation of the endothelial glycocalyx in clinical settings: searching for the sheddases. Br J Clin Pharmacol.

[20] Yen W, Cai B, Yang J, Zhang L, Zeng M, Tarbell JM (2015). Endothelial surface glycocalyx can regulate flow-induced nitric oxide production in microvessels in vivo. PLoS One.

[21] Biddle C (2013). Like a slippery fish, a little slime is a good thing: the glycocalyx revealed. AANA J.

[22] Henry CB, Duling BR (2000). TNF-alpha increases entry of macromolecules into luminal endothelial cell glycocalyx. Am J Physiol Heart Circ Physiol.

[23] Bruegger D, Jacob M, Rehm M, Loetsch M, Welsch U, Conzen P (2005). Atrial natriuretic peptide induces shedding of endothelial glycocalyx in coronary vascular bed of guinea pig hearts. Am J Physiol Heart Circ Physiol.

[24] Gouverneur M, Spaan JA, Pannekoek H, Fontijn RD, Vink H (2006). Fluid shear stress stimulates incorporation of hyaluronan into endothelial cell glycocalyx. Am J Physiol Heart Circ Physiol.

[25] Subramanian SV, Fitzgerald ML, Bernfield M (1997). Regulated shedding of syndecan-1 and −4 ectodomains by thrombin and growth factor receptor activation. J Biol Chem.

[26] Zuurbier CJ, Demirci C, Koeman A, Vink H, Ince C (2005). Short-term hyperglycemia increases endothelial glycocalyx permeability and acutely decreases lineal density of capillaries with flowing red blood cells. J Appl Physiol (1985).

[27] Kozar RA, Peng Z, Zhang R, Holcomb JB, Pati S, Park P (2011). Plasma restoration of endothelial glycocalyx in a rodent model of hemorrhagic shock. Anesth Analg.

[28] Torres LN, Sondeen JL, Ji L, Dubick MA, Torres FI (2013). Evaluation of resuscitation fluids on endothelial glycocalyx, venular blood flow, and coagulation function after hemorrhagic shock in rats. J Trauma Acute Care Surg.

[29] Mulivor AW, Lipowsky HH (2004). Inflammation- and ischemia-induced shedding of venular glycocalyx. Am J Physiol Heart Circ Physiol.

[30] Sillesen M, Rasmussen LS, Jin G, Jepsen CH, Imam A, Hwabejire JO (2014). Assessment of coagulopathy, endothelial injury, and inflammation after traumatic brain injury and hemorrhage in a porcine model. J Trauma Acute Care Surg.

[31] Chappell D, Bruegger D, Potzel J, Jacob M, Brettner F, Vogeser M (2014). Hypervolemia increases release of atrial natriuretic peptide and shedding of the endothelial glycocalyx. Crit Care.

[32] Koning NJ, Vonk AB, Vink H, Boer C (2016). Side-by-side alterations in glycocalyx thickness and perfused microvascular density during acute microcirculatory alterations in cardiac surgery. Microcirculation.

[33] Johansson PI, Stensballe J, Rasmussen LS, Ostrowski SR (2011). A high admission syndecan-1 level, a marker of endothelial glycocalyx degradation, is associated with inflammation, protein C depletion, fibrinolysis, and increased mortality in trauma patients. Ann Surg.

[34] Nelson A, Berkestedt I, Bodelsson M (2014). Circulating glycosaminoglycan species in septic shock. Acta Anaesthesiol Scand.

[35] Rahbar E, Cardenas JC, Baimukanova G, Usadi B, Bruhn R, Pati S (2015). Endothelial glycocalyx shedding and vascular permeability in severely injured trauma patients. J Transl Med.

[36] Ostrowski SR, Johansson PI (2012). Endothelial glycocalyx degradation induces endogenous heparinization in patients with severe injury and early traumatic coagulopathy. J Trauma Acute Care Surg.

[37] Johansen ME, Johansson PI, Ostrowski SR, Bestle MH, Hein L, Jensen AL (2015). Profound endothelial damage predicts impending organ failure and death in sepsis. Semin Thromb Hemost.

[38] Neves FM, Meneses GC, Sousa NE, Menezes RR, Parahyba MC, Martins AM (2015). Syndecan-1 in acute decompensated heart failure--association with renal function and mortality. Circ J.

[39] Zeng Y, Adamson RH, Curry FR, Tarbell JM (2014). Sphingosine-1-phosphate protects endothelial glycocalyx by inhibiting syndecan-1 shedding. Am J Physiol Heart Circ Physiol.

[40] Zeng Y, Liu XH, Tarbell J, Fu B (2015). Sphingosine 1-phosphate induced synthesis of glycocalyx on endothelial cells. Exp Cell Res.

[41] Chappell D, Jacob M, Hofmann-Kiefer K, Bruegger D, Rehm M, Conzen P (2007). Hydrocortisone preserves the vascular barrier by protecting the endothelial glycocalyx. Anesthesiology.

[42] Chappell D, Jacob M, Hofmann-Kiefer K, Rehm M, Welsch U, Conzen P (2009). Antithrombin reduces shedding of the endothelial glycocalyx following ischaemia/reperfusion. Cardiovasc Res.

[43] Chappell D, Hofmann-Kiefer K, Jacob M, Rehm M, Briegel J, Welsch U (2009). TNF-alpha induced shedding of the endothelial glycocalyx is prevented by hydrocortisone and antithrombin. Basic Res Cardiol.

[44] Chappell D, Dorfler N, Jacob M, Rehm M, Welsch U, Conzen P (2010). Glycocalyx protection reduces leukocyte adhesion after ischemia/reperfusion. Shock.

[45] Bruegger D, Rehm M, Jacob M, Chappell D, Stoeckelhuber M, Welsch U (2008). Exogenous nitric oxide requires an endothelial glycocalyx to prevent postischemic coronary vascular leak in guinea pig hearts. Crit Care.

[46] Jacob M, Bruegger D, Rehm M, Welsch U, Conzen P, Becker BF (2006). Contrasting effects of colloid and crystalloid resuscitation fluids on cardiac vascular permeability. Anesthesiology.

[47] Jacob M, Paul O, Mehringer L, Chappell D, Rehm M, Welsch U (2009). Albumin augmentation improves condition of guinea pig hearts after 4 h of cold ischemia. Transplantation.

[48] Haywood-Watson RJ, Holcomb JB, Gonzalez EA, Peng Z, Pati S, Park PW (2011). Modulation of syndecan-1 shedding after hemorrhagic shock and resuscitation. PLoS One.

[49] Mulivor AW, Lipowsky HH (2009). Inhibition of glycan shedding and leukocyte-endothelial adhesion in postcapillary venules by suppression of matrixmetalloprotease activity with doxycycline. Microcirculation.

[50] Marechal X, Favory R, Joulin O, Montaigne D, Hassoun S, Decoster B (2008). Endothelial glycocalyx damage during endotoxemia coincides with microcirculatory dysfunction and vascular oxidative stress. Shock.

[51] Eskens BJ, Zuurbier CJ, van Haare J, Vink H, van Teeffelen JW (2013). Effects of two weeks of metformin treatment on whole-body glycocalyx barrier properties in db/db mice. Cardiovasc Diabetol.

[52] Potter DR, Baimukanova G, Keating SM, Deng X, Chu JA, Gibb SL (2015). Fresh frozen plasma and spray-dried plasma mitigate pulmonary vascular permeability and inflammation in hemorrhagic shock. J Trauma Acute Care Surg.

[53] Torres LN, Sondeen JL, Dubick MA, Filho IT (2014). Systemic and microvascular effects of resuscitation with blood products after severe hemorrhage in rats. J Trauma Acute Care Surg.

[54] Broekhuizen LN, Lemkes BA, Mooij HL, Meuwese MC, Verberne H, Holleman F (2010). Effect of sulodexide on endothelial glycocalyx and vascular permeability in patients with type 2 diabetes mellitus. Diabetologia.

[55] Flameng W, Borgers M, Van der Vusse GJ, Demeyere R, Vandermeersch E, Thone F (1983). Cardioprotective effects of lidoflazine in extensive aorta-coronary bypass grafting. J Thorac Cardiovasc Surg.

[56] Borgman MA, Spinella PC, Perkins JG, Grathwohl KW, Repine T, Beekley AC (2007). The ratio of blood products transfused affects mortality in patients receiving massive transfusions at a combat support hospital. J Trauma.

[57] Holcomb JB, Wade CE, Michalek JE, Chisholm GB, Zarzabal LA, Schreiber MA (2008). Increased plasma and platelet to red blood cell ratios improves outcome in 466 massively transfused civilian trauma patients. Ann Surg.

[58] Pati S, Matijevic N, Doursout MF, Ko T, Cao Y, Deng X (2010). Protective effects of fresh frozen plasma on vascular endothelial permeability, coagulation, and resuscitation after hemorrhagic shock are time dependent and diminish between days 0 and 5 after thaw. J Trauma.

[59] Roche M, Rondeau P, Singh NR, Tarnus E, Bourdon E (2008). The antioxidant properties of serum albumin. FEBS Lett.

[60] Jorgensen KA, Stoffersen E (1980). On the inhibitory effect of albumin on platelet aggregation. Thromb Res.

[61] Richieri GV, Anel A, Kleinfeld AM (1993). Interactions of long-chain fatty acids and albumin: determination of free fatty acid levels using the fluorescent probe ADIFAB. Biochemistry.

[62] Pardridge WM, Mietus LJ (1979). Transport of steroid hormones through the rat blood–brain barrier. Primary role of albumin-bound hormone. J Clin Invest.

[63] Devaraj S, Yun JM, Adamson G, Galvez J, Jialal I (2009). C-reactive protein impairs the endothelial glycocalyx resulting in endothelial dysfunction. Cardiovasc Res.

[64] Deng X, Cao Y, Huby MP, Duan C, Baer L, Peng Z (2016). Adiponectin in fresh frozen plasma contributes to restoration of vascular barrier function after hemorrhagic shock. Shock.

[65] Peng Z, Pati S, Potter D, Brown R, Holcomb JB, Grill R (2013). Fresh frozen plasma lessens pulmonary endothelial inflammation and hyperpermeability after hemorrhagic shock and is associated with loss of syndecan 1. Shock.

[66] Nelson A, Statkevicius S, Schött U, Johansson PI, Bentzer P (2016). Effects of fresh frozen plasma, Ringer’s acetate and albumin on plasma volume and on circulating glycocalyx components following haemorrhagic shock in rats. Intensive Care Med Exp.

[67] Straat M, Muller MC, Meijers JC, Arbous MS, Spoelstra-de Man AM, Beurskens CJ (2015). Effect of transfusion of fresh frozen plasma on parameters of endothelial condition and inflammatory status in non-bleeding critically ill patients: a prospective substudy of a randomized trial. Crit Care.

[68] Guo C, Tsigkou A, Lee MH (2016). ADAMTS13 and 15 are not regulated by the full length and N-terminal domain forms of TIMP-1, −2, −3 and −4. Biomed Rep.

